# Dementia, Treatment Decisions, and the UN Convention on the Rights of Persons With Disabilities. A New Framework for Old Problems

**DOI:** 10.3389/fpsyt.2020.571722

**Published:** 2020-11-09

**Authors:** Kevin De Sabbata

**Affiliations:** Athena Institute, Vrije Universiteit Amsterdam, Amsterdam, Netherlands

**Keywords:** dementia, legal capacity, healthcare, autonomy, decisions

## Abstract

The UN Convention on the Rights of Persons with Disabilities has been at the center of considerable debate in the field of mental health. The discussion has caught up in particular after the publication of General Comment No. 1 in which the Committee on the Rights of Persons with Disabilities proposes a particularly radical interpretation of Article 12 of the Convention. Such a document has triggered skeptic and at times hostile reactions especially by psychiatrists, together with some positive comments. In this context, there is sometimes the tendency to focus only on the problematic aspects of the rights and support based model proposed by the CRPD and its Committee, forgetting that also “pre-CRPD” legislations on legal capacity present significant shortcomings. In this contribution I focus on the paradigmatic case of treatment decisions of people living with dementia with the aim to show how a number of provisions emerging from the CRPD and General Comment No. 1 can contribute to overcome the issues characterizing the traditional model of legal capacity and consent to treatment. First, I provide a brief overview of the provisions contained in the CRPD and General Comment No.1, summarizing the debate in this area. Then, I move to the case of treatment decisions of people living with dementia, analysing the main issues posed by the traditional model of capacity still characterizing European legislations. I will show how such problems and the solutions previously advanced by academics and practitioners resound in many ways with those identified by the CRPD and its Committee. In the second part, I analyse one by one the main provisions proposed by the CRPD and the Committee, studying how they can be applied in the area of treatment decisions of people living with dementia. In this context I point out the possible interpretations of the various provisions and their pros and cons, also referring to ongoing initiatives providing an insight on how such norms might work in practice.

## Introduction

Legal capacity is the ability of an individual to hold and exercise rights through valid legal acts (1). Those who have it recognized can legally decide for themselves, enter into contracts, buy a house, or decide who should inherit their assets; those who don't, cannot. At least since Roman times the law requires that, in order to be provided with such power, an individual must possess a certain level of intellectual and decision-making ability (mental capacity) (2). This is one of the most powerful and well-established gatekeeping concepts of legal agency. Therefore, it is not surprising that when someone tries to remove the gatekeeper there are strong reactions from those inside the gates.

This is what happened when the UN Convention on the Rights of Persons with Disabilities (CRPD) came into force. Article 12 of the Convention qualifies legal capacity as a universal human right which cannot be removed or limited because of a physical or mental disability [Articles 12(1) and 12(2)]. It requires that, if a disabled person struggles to make decisions, they should be given support through measures which respect their rights, will and preferences, rather than depriving them of legal capacity [Articles 12(3) and 12(4) CRPD]. In General Comment No. 1, the UN Committee on the Rights of Persons with Disabilities, the body in charge of monitoring the application of the Convention, has proposed a particularly radical interpretation of this provision (3). For the Committee, Article 12 requires the elimination from national legislation of guardianship arrangements mandating that a person, who is assessed as mentally incapable, should be stripped of the power to make valid legal decisions, which is transferred to a substitute decision-maker (e.g., guardian, trustee, or caretaker). Such an interpretation has triggered skepticism and, at times, hostile reactions. Traditionally, guardianship mechanisms have been justified by the necessity to make sure that especially people with a mental impairment are protected from choices that can result in self-harm or exploitation by others. Therefore, some have described the proposal to scrap them as something that “may end up hurting the very people it purports to help” (4) and risks “reversing hard won victories” (5). Moreover, it has been pointed out that not substituting the person would be impossible in cases of severe conditions like coma, lock-in syndrome, or very last stage dementia in which the individual seems to have no way to communicate with the outside world (6–8).

However, the old model is no nirvana. Substituted decision-making mechanisms exclude the person from the decision-making process and leave room for abuse (9). Moreover, mental incapacity can be difficult to evaluate and mental incapacity assessments present a high risk of returning incorrect evaluations of the person's decision-making ability (10, 11). Current mental capacity legislation tends to adopt a binary approach presupposing that there is a precise moment in which the person clearly appears incapable, and assumes that a person's decision-making ability is essentially dependent on their mental abilities (12). However, many conditions impacting mental capacity are progressive, and a person's decision-making ability is often influenced by the context of the decision (13–15). These problems emerge clearly in the case of people with dementia. Here, a person's cognitive abilities fade away gradually. Therefore, it can be difficult to distinguish between the moment in which a person with dementia is mentally capable of making a certain choice and the moment in which they are not (16–19). The person may often appear “just about capable, but not completely” or “probably incapable” but still be properly aware of a series of issues relating to the decision. Moreover, their cognitive abilities might be subject to fluctuation, so that on one day they seem completely lost and on another perfectly aware of the world around them. Therefore, declarations of incapacity with regard to a certain decision made on one day might have to be revised only a day later (20). Also, there are cases in which, once the person is put in a different, quieter environment or a decision is explained in simpler terms, the person becomes suddenly able to elucidate wishes they once seemed to have no clue about (21–23). In addition, it can be difficult for a guardian or trustee to ascertain what a person would have wanted in a certain occasion, or what is in their best interest. This difficulty is even more evident in fields, such as healthcare decision-making, in which, contrary to contracts for example, there are no objective criteria of economical convenience to assist the substitute decision-maker (24–26). In this context, one sees the value of many of the proposals for change advanced by the CRPD and its Committee, which have been perhaps too quickly discarded by some commentators.

Here, I will start from the paradigmatic case of healthcare decisions of people with dementia to show how the CRPD and General Comment No. 1 can provide a response to the shortcomings of the traditional model of legal capacity legislation. In doing this, I will proceed in four steps. First, in section 2, I will provide an overview of the content of the CRPD and General Comment No. 1, highlighting the discontinuities with the old model and summarizing the debate on the various provisions contained in them. Then, in section 3, I will move to the case of healthcare decisions of people with dementia, analyzing the issues posed by the traditional model of capacity still characterizing European legislations. I will show how the problems emerging in this context match with those identified by the CRPD and its Committee. In the last two sections, I will demonstrate how the main provisions proposed by the CRPD and the Committee can provide a better solution to the problems emerging in relation to healthcare decisions of people with dementia, and show how the problematic aspects of the CRPD model can be attenuated and are, in any case, less serious than those presented by the old model of decision-making. In this context, I will distinguish provisions which might indeed be utopian from those that can lead to a concrete improvement, and provide insights into ongoing initiatives emerging from healthcare and legal practice.

## Article 12 and General Comment No. 1

The UN Convention on the Rights of Persons with Disabilities (CRPD), was adopted by the UN General Assembly on the 13th December 2006 and entered into force on the 3rd May 2008. It is the first binding treaty reaffirming the rights of individuals living with “long-term physical, mental, intellectual or sensory impairments” [Article 1(2) CRPD]. Article 12 has a central position in the Convention architecture as it deals with the fundamental prerequisite for the full enjoyment and exercise of any right. It proposes a new human rights- and support-oriented model of legal personality and legal capacity.

Article 12(1) CRPD affirms that all disabled people have “the right to recognition everywhere as persons before the law.” Legal personality is the tool through which an individual or a group thereof are recognized as bearers of rights and duties. Therefore, through this norm, the Convention emphasizes that disabled people should be seen as *subjects* with rights, rather than *objects* of paternalistic interventions (27). Article 12(2) clarifies what this means concretely, affirming that “States Parties shall recognize that persons with disabilities enjoy legal capacity on an equal basis with others in all aspects of life.” Here, the term legal capacity refers to both the capacity to hold and exercise rights (so called *passive* and *active* legal capacity) (3). In this sense, Article 12 plays a crucial role in advancing the CRPD objective of promoting equality and non-discrimination of disabled people. The Committee emphasizes how legal capacity is indispensable for disabled people in order to exercise civil, political, economic, social, and cultural rights and be allowed to make fundamental decisions on their health, education or work (3). Disabled people, the Committee reminds, are the group whose legal capacity is most commonly denied throughout the world, as (mental) disability is traditionally regarded as a legitimate ground to deny the power to exercise rights, granted to all other citizens. Such a denial has in many cases led to disabled people being deprived of fundamental rights, such as the right to vote, marry, establish a family, determine care, and participate in economic life (3). Article 12 proclaims that such discrimination, and thereby rights deprivation on the basis of disability, is no longer legitimate, and that disabled people need to be treated like all other citizens and be awarded the same rights and power to exercise them. For this reason, the Committee on the Rights of Persons with Disabilities affirms that “under Article 12 of the Convention, perceived, or actual deficits in mental capacity must not be used as a justification for denying legal capacity” (3).

This vision shows a significant discontinuity with the traditional approach to legal personality, still adopted in the majority of legal systems in the world, in which the two concepts of legal and mental capacity are so conflated to be considered almost the same entity (3, 28). As pointed out by Richardson, the Committee's interpretation of Article 12(1) and (2) implies the abandonment of the traditional binary divide between legal capacity and incapacity on the basis of mental (in)capacity, and requires the abandonment of tests of mental capacity/incapacity contained in national regulations, which set out the cognitive prerequisites which, if not met, lead to the withdrawal of a person's legal capacity (29). There are three main types of mental capacity tests (30, 31). The first, the *status approach*, presumes that a person with a (mental) impairment diagnosis is, for this sole reason, incapable of making legal decisions. The second kind of test, the *outcome approach*, is based on the decision content, so that the person is judged mentally incapable, and hence deprived of legal capacity, if their choices seem irrational. Under the third kind of tests, the *functional approach*, a person's capacity is assessed on the basis of their actual decision-making related abilities. The abilities vary depending on the test and the legal system (31). For example, the functional test in section 3 of the Mental Capacity Act (MCA) 2005 of England and Wales focuses on the ability to understand, retain, use/weigh information, and communicate the decision, while the MacCAT-T, a test used in clinical practice to measure capacity to consent to medical treatment, focuses on a person's ability to understand information on their condition and treatment, reason on risks and benefits, appreciate the implications of their choices, and express a choice (32).

The first test is discriminatory because it relies on ungrounded assumptions, without checking if the person is really unable to make a certain choice. The second test is also discriminatory as it leads to disregard choices just because they are socially unacceptable (33, 34). As to the third test, scholars have initially considered it in line with the CRPD; indeed, it takes into account the nuances of the specific case and validates even decisions that others may consider irrational (35, 36). However, in General Comment No 1, the Committee clarifies that also the functional approach needs to be rejected as “it is discriminatorily applied to people with disabilities” (3). The Committee remarks that in all three tests, “a person's disability and/or decision-making skills are taken as legitimate grounds for denying his or her legal capacity” (3). Flynn and Arstein-Kerslake note how functional tests of mental capacity such as the one of the MCA “require either a ‘mental disability’or a finding of an ‘impairment of the mind or brain,’” and that “it is almost exclusively people with cognitive disabilities who have their legal capacity restricted on the basis of perceived decision-making skills” (37).

Another implication of this approach is that “States Parties must review the laws allowing for guardianship and trusteeship” and “replace regimes of substitute decision-making by supported decision-making” (3). According to the Committee, substituted decision-making, illegitimate under Article 12, are all measures in which (a) legal capacity is removed from a person even for a single decision, (b) a legal representative can be appointed against the person's will, and (c) the substitute decision-maker chooses according to the persons “objective best interests” rather than their will and preferences (3). Article 12(3) CRPD requires that, instead of these oppressive mechanisms, States Parties shall “provide access by persons with disabilities to the support they may require in exercising their legal capacity.” According to General Comment No. 1, supported decision-making is characterized by the fact that (a) the rights and preferences of the person are always respected, (b) support measures are proportional to the person's needs, (c) are available to everyone independently of income and severity of impairment, (d) do not hinge on mental capacity assessments, and (e) are always voluntary and provide safeguards against abuses (3).

With regard to who should deliver supported decision-making, the Committee states that such a mechanism “encompasses both informal and formal support arrangements” (3, 38). Informal arrangements consist of family members or friends voluntarily helping the person (39, 40). They have the advantage of being less costly and of involving individuals with whom the person is already familiar. Formal support arrangements would entail the appointment of a caretaker through a formal act (e.g., lasting power of attorney or authority decision) (38, 39, 41). Such kind of mechanisms can be useful especially when the person lives alone, as is the case with many older people nowadays. Moreover, being formally appointed, the support person can be subjected to regular scrutiny by a designated body. In both cases support tasks may be shared by a series of individuals. An important contribution may be also given by independent advocates, peer advocates, and peer-supporters who have the advantage of being external to the family, so more impartial and less inclined to exert pressure on the person in need of decision-making support (3, 42).

With regard to what support tools can be put in place, the Committee gives some (non-exhaustive) examples. The first possible means of support consist of providing accessible information, and reminding or explaining information in simple terms. Another way of supporting the person may be through the development and recognition of non-conventional methods of communication in case of individuals with difficulties in expressing themselves, like individuals with advanced dementia (3). The Committee emphasizes that supported decision-making should cover all possible decision scenarios pertinent to disabled people (3). Bach and Kerzner, describe support as a *continuum*, a dynamic process that follows the person in the evolution of the disability, articulated in three levels: “legally independent decision-making,” in which the disabled person is substantially able to make decisions on their own, “supported decision-making,” in which the person is assisted by someone that tries to help them in expressing their wishes, and “facilitated decision-making,” when, in case of significant disability, every attempt of communicating with the person is futile, rendering it necessary to reconstruct their will from previous declarations (43). With regard to this last level, General Comment No 1 indicates that where, after significant attempts have been made, it is not possible for the person to directly express their opinion, the decision should be made on the basis of “the best interpretation of will and preferences” (3). This means that those around the person and in charge of support should try to reconstruct the person's wishes on the basis of the person's behavior, previous statements, contextual elements or logical/presumptive reasoning, and identify a choice, which is as close as reasonably possible to what the person would want.

The interpretation of Article 12 as explained so far, has received at times harsh criticism. Dawson argues that the Committee's absolute position—that impairment in mental function should never be a ground for denial of legal capacity—is impossible to support as “contrary to the fundamental principles of virtually any sophisticated legal system, whose doctrines will—rightly—be saturated in mental concepts, such as intention, knowledge, foresight, and the ability to process information.” Reasoning on these grounds, he affirms, may be highly advantageous to the person, preventing inappropriate punishment, or triggering entitlements to additional support (6). Freeman et al. note that “there are times when informed consent is not possible […] and must be superseded, particularly where life is at risk.” They insist that, in case of coma, severe infectious disease or neurological conditions, not making an “exception” to the recognition of legal capacity and administering treatment without consent would lead to harm to the person or others and actually infringe upon their right to health or dignity (5). Appelbaum laments that under the Committee's interpretation of the CRPD, “elderly persons with dementia, no longer able to care for their own needs but unwilling to accept management of their finances, health, or living situations by a guardian, could not be compelled to do so. People intending to end their lives as a result of major depression could not be hospitalized against their will, nor could persons suffering from psychosis who are refusing to eat because they believe their food is poisoned” (4).

The members of the Essex Autonomy Project maintain that the Committee's interpretation goes beyond the text of the CRPD and that the Convention “does not actually say that substitute decision-making should be abolished.” They argue that functional tests of mental capacity are not discriminatory, but they simply treat in a proportionally different way situations which are made different because of the person's particular mental abilities (7). According to Scholten and Gather, the Committee's model of legal capacity does not guarantee the conditions for the person to correctly exercise their autonomy, consciously assessing which treatment option will be most consistent with their well-being and their understanding of what is best. In addition, they point out that the support-centered model proposed by the Committee poses a higher risk of undue influence, as it can be difficult to distinguish between assistance in self-expression and diversion from a person's will, and that providing support can be very costly and time-consuming (44). Dawson affirms that the Committee fails to recognize the potential difficulties in determining a person's “will and preferences” when they have left no clear expression of their prior views, and it also fails to clarify when “supported” becomes “substitute” decision-making in the reconstruction the person's likely wishes (6). In this regard, Scholten and Gather note how the Committee's interpretation of Article 12 CRPD limits possibilities for advance care planning. Indeed, advance directives, the main instrument for care planning, are by definition a tool for mentally capable individuals to set out their care wishes for when they will be mentally incapable, so one wonders what sense they would make in a system that does not distinguish between capacity and incapacity (44).

## Legal Capacity, Healthcare Decisions, and Dementia

A number of the concerns expressed by critics of the Committee's interpretation of the CRPD are understandable. As in all revolutionary regulations, also in the case of the CRPD and General Comment No. 1, there are places in which progressiveness and commitment to social change might appear to have trespassed into utopia, and, in the implementation phase, some proposals might need to be partially adapted. Practitioners and mentally disabled people know by direct experience how mental impairments create concrete issues, which impact a person's ability to make decisions and cannot be resolved just by proclaiming that their legal choices are valid whatever their mental abilities (45–47). The concern that legally validated choices put in place in a state of diminished mental capacity may lead to harmful consequences for a person is justified and serious (48, 49). However, a number of these objections seem to derive from a misinterpretation of the Committee's statements, which still tend to be read in light of old categories. In addition, a number of the shortcomings that critics identify in the model proposed by General Comment No. 1, also characterize current legal capacity regulations. In this sense, General Comment No. 1 proposes solutions that, though not perfect, still represent a step forward. Article 12 CRPD and General Comment No. 1 offer potentially valuable responses to serious and longstanding problems characterizing current approaches to legal capacity. Such problems emerge particularly clearly in relation to healthcare decisions of people with dementia.

Dementia currently affects around 50 million people worldwide, of which around 10.5 million live in Europe (50, 51). They are 90–98% older individuals (over 65 years of age) (52). Dementia is an umbrella term which refers to several types of conditions affecting the cognitive functioning of an individual. Such conditions are characterized by a progressive and significant decline in one or multiple cognitive domains such as learning and memory, language, executive functioning, complex attention, perceptual-motor, and social cognition. For a formal dementia diagnosis to be reached, the deficit must interfere with independence in everyday activities, not occur exclusively in the context of delirium, and cannot be explained by the presence of other mental health disorders (53). Moreover, dementia may provoke changes of personality and opinions, and challenging behavior such as agitation, restlessness, aggressiveness, non-cooperative attitude, or apathy (54). Also, due to their advanced age people with dementia are often in need of various kinds of medical treatment and associated support. However, due to their condition, they could be seen as lacking the intellectual ability required to make conscious treatment and care choices, understand information given in conventional ways and remember their doctor's explanations (13, 55–57). Moreover, in the final stages of the condition, the person may be unable to talk and move, and not be able to communicate their wishes and feelings (58). Therefore, questions of mental capacity can be pretty frequent in relation to healthcare choices of people with dementia. However, current rules in this field can be quite difficult to apply.

A person with dementia's reasoning abilities deteriorate gradually and fluctuate on a daily basis, so that it can be difficult to establish the exact moment in which the individual becomes incapable (13, 20). Moreover, the condition impacts the different cognitive abilities unevenly, so that people with dementia might be unable to grasp a certain implication of the decision, but appear perfectly aware of other aspects of it, so that they are still able to give meaningful indications which need to be taken into account, though it is not always clear what weight they deserve (20). Because of their condition, they might also adopt unconventional ways of expressing their opinions (23, 59, 60), following modalities which leave doubts as to whether they really do not understand a certain issue, or are just voicing it in a way that we do not understand. In addition, people with dementia are very much influenced by their surroundings (e.g., background noise), so that one can be unsure as to whether they really are mentally incapable or only distracted by external stimuli (23, 61, 62). Finally, doctors, relatives or surrogate decision-makers also report difficulties in making decisions on the basis of people with dementia's best interests or presumed will when they are in the final stages of the condition (24–26). By looking at the case of healthcare decisions of people with dementia, five main shortcomings of the current approach to legal capacity regulation emerge, which I will discuss here. They are, the difficulty in applying the capacity/incapacity distinction, the narrowness and discriminatory nature of mental capacity tests, the potential paternalism and risk that the person's opinions are disregarded, the lack of mechanisms to account for and deal with external barriers to healthcare decision-making, and the malfunctioning of current mechanisms for surrogate decision-making.

The first issue derives essentially from the fact that, while dementia is a progressive condition, current legal capacity regulations tend to adopt a binary approach based on the sharp distinction between mental capacity and incapacity. Therefore, establishing when a person with dementia becomes mentally incapable is a bit like squaring a circle. Clough notes that the current binary approach to legal and mental capacity is both under-inclusive, as it does not give any help to those that are “just about capable,” and over-inclusive, as it takes away the power to make a certain decision from people who, despite the declaration of incapacity, might still have a partial ability to make choices and express opinions on some aspects of the choice (15). Because of the difficulty of “fitting” such a binary category in the case of dementia, even experienced assessors, or surrogates may have doubts with regard to determinations of capacity or incapacity of the person (11, 16, 63). Quantitative experimental studies involving doctors confirm how they are frequently in disagreement with each other especially with regard to the capacity of individuals in the initial and mid-stages of dementia (18, 19, 57, 64, 65). This uncertainty might result in doctors and carers often prematurely attributing a person's statements to mental incapacity and wrongfully discarding them, because they are forced to interpret a patient's mental ability based on a binary scheme.

This risk that a person's statements are too easily discarded is made worse by the fact that mental capacity tests, including the most progressive ones such as the functional test in section 3 MCA 2005, disregard the importance of a series of non-cognitive mechanisms which play a crucial role in the way people with dementia make choices. In this regard, they have been criticized for being “hypercognitivistic” (66, 67). Indeed, they focus on abilities related to logical understanding, and retaining and weighing of information on the nature and consequences of a decision, and the capacity to express a choice based on the consideration of such information (68). However, these parameters do not reflect how decisions are made in real life. As Quinn puts it “most of us, most of the time, both think and act irrationally” and often our ability to decide is really determined by the environment in which we act, rather than our brain (27). Referring to empirical studies in psychology and behavioral economics Wright points out that often decision-making is distorted by cognitive biases and irrational considerations (69). In this regard, current functional tests of mental capacity overlook important dynamics such as feelings, identity, values, practical reasoning, and narrative (68, 70–73). Considerations pertaining to these domains may be more prominent than logical reasons in making choices, so that an individual may decide to give them precedence over rational arguments. After all, don't we mainly make choices because of emotional factors, because we are afraid of pain, or because we are attached to certain ideas or ideologies? This may be even truer in the case of people with dementia as, because of the deterioration of their cognitive abilities, they tend to rely on other mental resources to make decisions, being guided more by their emotional-irrational sphere (74).

In this sense, the current approach to legal decision-making puts people with dementia automatically in a position of disadvantage and results discriminatory. Indeed, legal capacity legislation and models of healthcare decision-making are based on the principle according to which an adult must be presumed to have capacity unless they are proven to have a mental condition and that this impacts their judgement (66). This means that people with no signs of mental impairment will not be subject to the “hypercognitivistic” capacity tests in use in modern legal systems, remaining free to make decisions on the basis of their emotions, fears, and eccentric wishes. Only mentally disabled individuals, like people with dementia, run the risk of being questioned in their decisional ability, and have to demonstrate that they satisfy an unrealistic standard that very few of their fellow human beings would meet in usual life situations. Therefore, the General Comment No. 1 critique of mental capacity tests is echoed also in previous literature on the capacity to make healthcare decisions and on healthcare choices of people with dementia. In this regard, the case of people with dementia shows that the claim, advanced by critics of the Committee (5–7), that functional tests of mental capacity are neutral and not discriminatory is not sustainable. If the possession of the intellectual faculties required by such tests is indispensable for a correct exercise of the person's autonomy, then it should be applied to treatment and care decisions made by all people, even if they do not have a diagnosed mental impairment. This, however, might risk declaring invalid a series of decisions which are now normally accepted.

Furthermore, the very existence of the legal category of mental incapacity increases the risk that an individual's expression of will, such as that of people with dementia, can be disregarded. Current regulations are based on the assumption that, if a person's cognitive impairment has a tangible impact on their decision-making ability, it is right to strip them of their legal capacity. Doctors, carers and relatives of people with dementia know that such a moment is due to arrive soon, and the possibility that a certain behavior is “the product of dementia,” rather than the person's “genuine will” is constantly in the back of their mind, making them more prone to ignore the person's opinion (75). Many practitioners and carers still frequently equate a diagnosis of dementia with incapacity to decide on care (13, 69, 76–78). Surveys and experimental quantitative studies performed on individuals with mild to moderate dementia show that these individuals risk being judged totally or partially incapable to make a certain decision significantly more often when the assessment is made by their doctors than when it is carried out by external researchers (18, 57, 64, 79). The Leeds Christian Council on Aging complains that “it is too easily assumed by the decision makers in providing care (as well as generally) that people with dementia are incapable of making choices and taking decisions […] thereby ‘de-humanizing’ them” (80). This frustration emerges also in statements by people with dementia. A person in the early stages of this condition, cited in a qualitative study by Phillips, remarked: “the reality is that when diagnosed with Alzheimer's, we are immediately discounted; our views are discredited because of the disease” (81). Taylor, in the memoir of his experience with Alzheimer Disease, laments that he is often not taken seriously: “my behavior is treated as something apart from me. ‘It’s not him, it's the disease'” (82).

However, in reality, in a number of cases, the inability of people with dementia to articulate choices on care and treatment derives not so much from their cognitive impairment, but rather by the environment surrounding them or the way in which people communicate with them. The effectiveness of information disclosure on treatment and care options to people with dementia is often jeopardized by the fact that it happens in noisy environments, as hospital wards often are, or while parallel conversations are taking place in the same room; a situation which makes it difficult for the person with dementia to absorb information (23, 61, 62, 83). Cowdell reports how care staff often tend to “bombard” their patients with information without giving them the time to process it and using, in some cases, quite dismissive and even aggressive manners (84). Of course, a person's impairment does have an impact on their decision-making ability. Because of memory loss, they may not remember crucial elements relating to a proposed treatment or care options or facts of general life potentially relevant to the decision (26, 85, 86). In addition, people with dementia may not always realize that they need care or treatment (87). However, at least in the early and intermediate stages of the condition, these sort of problems could be avoided or mitigated through communication strategies and mechanisms directed to reminding the person of things they may not recall, or explaining treatment information in simpler terms or by rectifying misunderstandings, or planning conversations with the person in moments in which they are sharper (78, 88–90). Unfortunately, the majority of national legislations do not provide mechanisms to assist a person in overcoming the obstacles created by their condition, and rules that require active help before declaring a person incapable (91). In this regard, according to empirical studies on capacity assessments in clinical settings, medical, and legal professionals tend to assume that they have to limit their assessment to what they see at first sight, without asking themselves what would happen if the person is helped in understanding the reasoning and implications of the proposed medication (18). Yet, a number of medical practitioners, scholars, and individuals, that have been close to people with dementia, affirm that if the right conditions are in place, and the person is really listened to, they are often able to understand the substance of proposed medical treatments and express articulate decisions on their care and treatment until advanced stages of their condition (57, 92, 93). In this regard, the CRPD's emphasis on a model centered on supported decision-making can lead to a welcome improvement on the extent to which the person is enabled to express their opinions directly.

Nevertheless, even with all support possible, there does come a time when a person with dementia cannot communicate anymore with the outside world and it is inevitable to make decisions for them. For these cases, national regulations provide a series of surrogate and substituted decision-making mechanisms. However, literature and experiential accounts of healthcare professionals, relatives, and surrogates, show how such instruments are often very difficult to apply. Under current systems, surrogate decision makers can be appointed, depending on the jurisdiction, either through a voluntary act (power of attorney), a guardianship order, or be legally identified among family members and next of kin. Two main paradigms of surrogate decision making exist: best interests or presumed will (also known as substituted judgement). The former looks at what would be “objectively better” for the person in light of supposedly factual considerations (24). The presumed will standard requires the identification of how the person, if capable, would have made the decision, trying to infer their hypothetical wishes from elements such as previous statements of the person, their values, their personality, habits or previous similar decision scenarios, and corresponding choices (94). In relation to best interests, especially regarding intimate choices, such as those on care, it could be difficult to establish what is “good for a person” in absolute terms (24). In this regard, contrasting views can be presented by doctors, relatives or other people around the individual (25, 26). Moreover, the concept of best interests seems to presuppose that someone knows better than the person what has to be done and can impose it on them. For this reason, the best interest model appears rather paternalistic. On the other hand, reconstructing the presumed will of the person might be far from easy, for example because of the cognitive fluctuations and changes of opinion, which may leave contradictory signs of what their real wishes are (16). Moreover, relatives and friends often have very poor and incomplete knowledge of a person's intimate care intentions: this can be a significant obstacle to the correct reconstruction of such intentions (95).

Another possible legal tool in this case is advance directives, through which a person can set out their care wishes for the time in which they will be mentally incapable due to dementia (96). However, such declarations often are issued years before they are due to be applied and the person has no precise knowledge of the kind of medical treatments or care options among which they would have to choose (95, 97, 98). Therefore, such directives can be vague and difficult to interpret. In addition, the person can change their mind either because of direct experience of living with dementia, the different world that their cognitive impairment depicts in front of them, or simply because of the passing of time. Therefore, after developing dementia and being declared mentally incapable, they may express opinions or enact behaviors which are in contrast with their anticipated will (12). In these situations, doctors, family members, and carers find themselves confronted with the dilemma of whether to follow the advance directive and ignore the current will of the person, or ignore the advance directive and follow the person's current wishes. In the academic debate, positions are split between those who are in favor of the former solution (99) and those who support the latter (100, 101). However, both options are at a certain extent unsatisfactory (102). On the one hand, giving precedence to the person's advance directive leads to ignore statements which have been expressed in light of direct experience, and might be seen as a paternalistic way in which a mentally capable individual is allowed to overrule their self, once they are affected by dementia. On the other hand, giving precedence to the current wishes of the person with dementia could be seen as a diversion from the life plan that a person has set out for themselves, exercising their right to autonomy in a moment in which they could elaborate a well-thought through vision of their values. In addition, also the contemporary statements of the person with dementia can be unclear and difficult to understand.

## The CRPD, Dementia, and Healthcare Decisions

The model of mental capacity and legal decision-making proposed by Article 12 CRPD, appears to avoid or attenuate many of the problems explained in the previous section. First of all, it skips the problem of having to apply to a progressive condition like dementia the binary distinction between mental capacity and incapacity. Under Article 12 CRPD, this divide does not exist anymore, there is no point at which a person becomes mentally incapable, but only a process of change which the individual undergoes due to their condition and is accompanied by a constant evolution of support mechanisms, which adapt to the person's changing needs on a spectrum that goes from the simple explanation of information in more accessible terms to the reconstruction of the person's will and preferences. The person's power to make legally valid decisions is not subordinated to their level of cognitive abilities. This means, first of all, that the person will not be subject anymore to mental capacity assessments under often too narrow, “hypercognitivistic,” pre-set criteria. So, by interpreting their statements and supporting them in expressing their will as much as possible, it will be possible to take into account a broader set of factors relating to a person's irrational and emotional sphere, and to include identity, values, practical reasoning, and narrative considerations. This model of decision-making would be more inclusive toward unconventional ways of reasoning, communication, and sense making. In addition, by uncoupling the validity of a person's decision from their mental capacity, General Comment No. 1 radically excludes that a person with an impairment like dementia can be deprived of legal capacity. This is expressed as an absolute and uncompromising position. In this way it attenuates the risk that healthcare and legal professionals, family members or carers, set aside a person's wishes too soon and discard them as invalid. Indeed, General Comment No. 1 takes away from the table the possibility of legal capacity deprivation altogether. Now healthcare professionals, carers, family members or proxies are clearly told that, no matter what are a person's impairment and cognitive abilities, the former must listen to what the latter has to say and implement their wishes.

This message is made even stronger by the ban on forms of substituted decision-making and old-fashioned guardianship mechanisms in which a guardian, trustee or caretaker has the power to make decisions impacting a person's legal sphere without their explicit consent. Frequently, guardianship arrangements have been the means for abuse and oppression against disabled and older people, including those with dementia (9, 103). In many countries, the rigidity of guardianship regimes has been attenuated, giving some more weight to a person's view. However, the possibility of stripping the person of their decisional power and making decisions without involving them still remains (104). This creates a situation in which the guardian, trustee, or caretaker is authorized and even required to trump the will of the person. In a context in which the person, because of their condition or advanced age, might be in a vulnerable situation, there is a risk that the guardian may overstep the mark and start ignoring the person's wishes, even when their requests, once seriously considered, are sensible ones. This is even more dangerous if we remember that, here, we are dealing with a number of decisions on everyday care arrangements, or therapeutic procedures, which are made in the secrecy of private and care homes, or hospital wards, and which are difficult to monitor for overseeing authorities. The Committee on the Rights of Persons with Disabilities responds to this issue, clarifying that, under the CRPD, no decisions are ever admissible which are not centered on the will of the individual.

In addition, the universal support model proposed by the CRPD and promoted by the Committee addresses the issue of external barriers which prevent people with dementia from participating in decisions on their health care. Indeed, the Convention provides a platform, supported decision-making, through which the person can be actively helped to overcome such barriers, and the conditions can be created for them to be involved as much as possible. Recent research projects provide some indications of supportive measures which can help people with dementia to make decisions on their care and medical treatment (90). For example, a successful support strategy reported by a series of studies is that of narrowing down alternatives relevant to a certain choice, as individuals with dementia tend to be confused and overwhelmed when presented with too many options (21, 105–107). Other generic support means consist of explaining concepts and information in a simpler way which matches with the person's needs (e.g., speaking loudly and clearly, with short phrases, avoiding words the person does not understand) (105, 106), giving the person time to process information and not rushing them (21, 62, 105–109), clearly defining the question under discussion (108), and reminding the person of certain information (21, 105, 110). Sinclair at al. also stress the importance of prompting techniques, such as giving cues or using words like a “little key” to unlock particular memories (105). The use of visual illustrations, aids, and props appears to be also useful in helping a person understand information and making a choice (21, 109). In another study, Sinclair et al., show how it is possible to support the person in making decisions by employing “Augmentative and Alternative Communication” (AAC) techniques combining multiple sensory modalities (e.g., aural and visual), observing facial expressions, body language, sounds made by the person, or eye contact, and using structured approaches to communication (106). Support can also come from IT decisional aids sending reminders to the person or guiding them through the decisional process (111, 112). Finally, just creating a quieter and tidier environment and encouraging the person to express their wishes (21, 62, 109), or defending them from others' disempowering behavior (105) may be important means of support.

By indicating this kind of support measures as the only admissible legal capacity measures, the Committee makes a powerful statement in the attempt to induce healthcare professionals, family members, and carers to actively engage in promoting the person's will at a greater extent than they have done in the past. National provisions, such as section 3(2) MCA 2005, already establish that “a person is not to be regarded as unable to understand the information relevant to a decision if he is able to understand an explanation of it given to him in a way that is appropriate to his circumstances.” However, practitioners and people in the relationships network of people with dementia tend to ignore this intermediate step, rushing to the declaration of mental incapacity (10, 113). Now that, under the CRPD and General Comment No. 1, this kind of declaration is not admissible anymore, they are being compelled to try every possible mechanism to explain information to the person and create the best possible conditions for them to express their will.

However, this radical approach leaves open a series of questions. The first open question, as suggested by some, is that in a series of cases a mental capacity assessment could be inevitable (4, 6, 7). One could say that also in a system entirely based on supported decision-making there is the need to assess the person's reasoning abilities, in order to provide adequate means of support. In addition, despite all efforts, supported decision-making practices are not always successful (8, 114). There are cases in which a person might make, or be “trapped” into making, a decision which is harmful for them and would not have made, if they had previously received adequate support, or even despite having been duly supported. There may also be situations in which it is necessary to administer medical treatment without the person's consent or despite their refusal in order to handle crises, stabilizing, and putting them in the condition to participate in decisions and be supported, or to guard their physical integrity or manage situations of emergency in which it is not possible to talk adequately with the person and guide them in choosing for themselves (4, 6). The remedy to all such situations was traditionally the binary concept of incapacity, which used to act as a ground for invalidating harmful decisions and administer treatment without a person's consent in situations in which otherwise their physical or psychological integrity would have been at risk. But in the CRPD system, in which this category is not admissible anymore, there is the need to find alternative mechanisms which take on this function.

With regard to the first issue, in reality, in order to provide adequate support, a capacity assessment is not strictly necessary. Supported decision-making responds to practical needs of the person which are manifested through concrete situations and behaviors. For example, if the person struggles to remember things, than they will need someone reminding them, if they struggle to find words, they will need help through various communication strategies. In establishing what to do in such circumstances, a mental capacity assessment based on a legalistic standard will not be of much help. Rather, there will be the need to know what specific communication strategy should be put in place, or what environmental adaptations would help the person to actively take part in conversations on their care. In this context, what helps more than mental capacity tests, is the knowledge emerging from studies, guidelines, expert advice of speech therapists or psychologists, and the experience of nurses, carers and family members.

Studies and pilot projects on treatment and care decision-making for people with dementia show how it is possible to organize support around the person without the need of a mental capacity assessment. A team of German researchers has developed the CODEMamb tool, which allows for investigating the needs for (conversational) support and adjustments of a person with dementia by observing their communication behavior and resources (89). In a Norwegian pilot study investigating shared healthcare decision-making involving people with dementia, Smebye, Kirkevold, and Engedal identified different levels of support needed, which present with the development of a person's impairment, but in a fluid way, just adapting to the person's conditions and reactions. Therefore, the support provided evolves from the autonomous decision-making stage, in which the person just needs a few explanations on the what, why and how of the decision, to a shared decision-making level, in which the decision is made in collaboration with professionals and informal carers who act as supporters and are allowed by the person to compensate for their lost abilities, to a delegated decision-making arrangement or a stage in which due to communication difficulties the supported decision-maker uses information and insights gathered in the previous phases to make choices for the person (21). The impression is that current approaches to legal capacity have been excessively and unrealistically relying on mental capacity assessments, which tend to be seen as the panacea to all problems (115, 116). Whenever the person has a care and support need, they are put through an assessment which at times is not even followed by the actual service provision, giving the impression that hospitals and care services are more interested in assessing and controlling people, than helping them. Therefore, General Comment No. 1, by excluding mental capacity assessments, emphasizes, once again, the centrality of support services rather than bureaucratic procedures.

However, one further objection that could be brought forward is that, in the current system, mental capacity assessments also function as a guarantee against the risk that people are deprived of their legal capacity without a legitimate justification or with opaque procedures, and to make sure that the adopted legal capacity measures are appropriate in relation to the persons' decision-making ability. Mental capacity assessments are often presented as transparent and objective mechanisms to scientifically evaluate a person's reasoning abilities (32, 117). However, as we have seen, they offer only a very partial representation of what making decisions means in practice. They are based on value laden assumptions influenced by the liberal tradition at the basis of private law regulations in most Western countries, which identify rationality and the ability for logical reasoning of a self-standing individual as the fundamental requirement to be considered a citizen and be recognized legal agency (27). As already pointed out, such assumptions do not seem to reflect the way in which the majority of people, even without a mental impairment, make choices. In addition, assessing the ability of a person to understand, weigh, retain or appreciate information regarding a certain choice, is not a mechanical process, but is highly subjective and leaves room for prejudices and false assumptions, especially in relation to conditions like dementia (18, 57, 64, 79). The CRPD and the Committee try to anchor (supportive) legal capacity measures to more factual elements observable without the need for a controversial capacity assessment, such as communication difficulties and gaps in mnemonic recollection.

Of course, the problem remains of making sure there is a test against which we can double check both that healthcare professionals or next of kin have correctly established how to assist the person in making decisions, and that guarantees to them that, once they have rigorously followed such a set of clear steps, they are not at risk of being sued for negligence. This problem can be solved, rather than with a capacity assessment, by developing clear professional guidelines on supported decision-making. This is also the way in which negligence cases are handled in many legal systems. In order to judge whether a healthcare professional has acted diligently or negligently, the judge checks whether they have followed the best advice elaborated by their scientific and professional communities, which is enshrined in professional guidelines issued by authoritative bodies and constantly updated in light of practice evolution. Such guidelines can be developed also for supported decision-making in healthcare choices, and some attempts have already been made which can be relevant for people with dementia (118, 119). The German Society of Gerontology and Geriatrics, the German Association for Psychiatry, Psychotherapy and Psychosomatics, and the German Neurological Society, in collaboration with the *Alzheimer Gesellschaft*, have recently issued a document containing guidelines precisely on how to support people with dementia in deciding on medical treatment, which can be used to ascertain whether medical and care staff have acted diligently in helping the person to make therapeutic choices (120). These guidelines are praiseworthy also because people with dementia and carers have been involved, in the development phase, through their most representative national organization.

With regard to the problem of legal invalidity and of having a ground for dealing with (self)harmful choices or allowing for involuntary treatment, the CRPD does not eliminate the possibility of canceling decisions made for example in a moment of crisis, and that the person would have not made otherwise. It only handles them on a different dogmatic platform. To begin with, as remarked by Arstein-Kerslake and Flynn, the CRPD does not exclude the legal remedies present in all private law legislations, that permit invalidation of declarations which are the result of misrepresentation (121). Moreover, the function of clarifying the will of the person and setting aside statements which are the product of incorrect understanding, may be included among the supporter's role of interpreting and reconstructing the person's wishes seen above. This also applies to those situations in which the person does not consent to a treatment which would be necessary to protect the person's life or physical or psychological integrity. The claim brought forward by critics of General Comment No. 1, according to whom the model identified in the document would simply leave suicidal individuals or people unable to understand the consequences of their choices at the mercy of the self-harmful actions, is based on a misunderstanding of the true meaning of the CRPD and the Committee's interpretation. The aim of Article 12 and General Comment No. 1 is not to abandon disabled people to themselves, quite the opposite. The CRPD strives to give more help and better protection to these individuals. This protection, however, is established through supported decision-making, rather than oppressive mechanisms based on legal capacity deprivation.

In this context, individuals in charge of supporting a person may enter into a dialectic dynamic with the latter, inviting the individual with dementia to reconsider facts or interpretations which might have been affected by misunderstandings (e.g., the person considers alive people who are dead, or counts on medications which are not viable) (122). In this case, the role of the support person might also include saying “no” to the beneficiary, or urging to postpone a certain decision to a later time in which the person is more alert (20), or proposing to disregard statements which are in contrast with the usual view of the person on a certain matter. Of course, this dialectic dynamic has to be intended not as an occasion to challenge the person's beliefs or decisions, but only to rectify factual misunderstandings or manage situations of distress. In a case study, Zinkler and I have analyzed a case of supported decision-making of a man with schizophrenia and life-threatening pneumonia, who refused both antipsychotics and antibiotics. The carers and guardian, after attempting to persuade the person, and in consideration of the fact that he expressed the will of remaining alive, interpreted his will in the sense of respecting the person's refusal of antipsychotics and administering the minimum necessary of antibiotics in order to eliminate the risk of death of pneumonia (123).

Therefore, in the system proposed by General Comment No. 1, the functions of resolving contradictions and setting aside declarations made by the person when “*non compos sui*” are transferred from the level of legal capacity and validity, to that of interpretation. This is not just a cosmetic change. First, it moves away from a mechanism for validating legal decisions based on mental capacity and controversial judgements on what is needed to make decisions, to a coherentist approach, which evaluates a person's statements in light of the evolution of their thoughts and values. In this way, it avoids that assessments on the person's inability to understand, retain or weigh information and communicate decisions, may hide a prejudiced judgement. Instead, the Committee's approach proposes a system which starts from the person's own values, independent of how eccentric they appear to be. In addition, in the interpretation of the person's will on care, the person themselves plays a role. They can participate in the effort of clarifying their statements, following a mechanism also based on negotiation and the central role of the person's input. Adopting this approach also allows to more correctly assess whether the support provided has succeeded in empowering the person and enabling them to make decisions for themselves. Indeed, looking at the consistency of decisions with the person's values, practical reasoning, expressed narrative and emotions, and discussing with them on how much a certain decision represents them, would return a more faithful, prejudice-free evaluation than a mental capacity assessment. Regardless, considering the CRPD commitment to promoting equality and non-discrimination of disabled people, allowing for more “hands-on” mechanisms of protection and support evaluation would be inconsistent with the spirit of the Convention. Indeed, if we really believe that disabled people should be treated like all other citizens with regard to the validity of their decisions, we need to do the same when dealing with the harm potentially deriving from such choices. Also, in the case of individuals with dementia, after all viable support tools and discussions have been put in place, we have to allow the person to face the negative consequences of their choices if they are convinced to go down a certain route. Of course, moving away from a system focused on mental capacity assessments, and embracing a coherentist approach focused on dialogue and the interpretation of the person's statements will require time and a significant change in the mentality of practitioners. However, we need to get there in the end. Continuing to assess decision-making skills and the effectiveness of supported-decision making against unrealistic, hypercognitivistic capacity standards established a priori would still mean forcing people with dementia into a model of decision-making which does not reflect what happens in everyday reality and use support to give them something which might be not in line with what they truly need.

## Dementia and the CRPD Model: Potential Issues

Other objections, brought forward regarding the support centered model proposed by the CRPD and the Committee on the Rights of Persons with Disabilities, are that providing support is costly, places additional tasks on already overworked healthcare professionals, and that it can easily turn into forms of undue influence. With regard to the first issue, we always have to bear in mind that we are dealing with the need that doctors, nurses, family members, and carers talk with people with dementia. Communicating with their patients and assisted family members is already a duty of these individuals. This duty is not specifically related to healthcare and legal decision-making, but more generally to the right of every person, and of disabled or older people in particular, to have adequate care and support. The fact that, in this historical moment in western societies, the state is disengaging from its obligation to provide care to all their citizens, including those who can count on less financial and human resources within their family, does not mean that neglect and lack of humane care should be accepted or justified. In addition, shared and supported decision-making projects in hospitals and care homes in Italy (23), The Netherlands (23, 108), Norway (21), and Australia (105, 106) show that it is possible to provide support to the person with dementia at very limited costs by using the people within the person's circle of relationships. Besides, as remarked by Flavin, herself a person with dementia, the cost of providing support for decision-making is often outweighed by the benefits consisting of the person remaining engaged and feeling more fulfilled and confident, thus requiring less care and having a relatively less problematic disease trajectory (124). Of course, some people might not have a support network, or have relatives who are not available to act as support people or who act disruptively in the support relationship. In these cases, precious help can come from charities and associations of people with dementia, who can provide advocacy services and peer-support networks for people living alone (125, 126). As part of the effort to implement the CRPD, national governments should ensure that such organizations are well-supported and appropriate services exist for people who do not have an extensive support network. In addition, it would be advisable that the person puts in place, in good time, a formal “support agreement” establishing a clear distribution of tasks for the members of their network and, if needed, entrusting external individuals or organizations with the responsibility to support them in making decisions (105).

With regards to the risk that support turns into forms of undue influence, this is certainly something to take very seriously. Explaining information, guiding conversations, and simplifying decisions by reducing the number of alternatives are all highly subjective exercises, in which it is impossible to be totally neutral (127, 128). A support person influences the person with dementia simply by choosing the topics and timeframe of the conversation, or by the way in which they present issues. Flavin shows how often undue influence and paternalism occurs in situations in which the person is spoken to, supposedly, in a compassionate, gentle and supportive tone, but in reality, the support people dictate to them what to do and when (124). Moreover, due to the dynamics of dependency inherent in care relationships, it is often the support person who retains the power to decide if, and if so when, to initiate support (21, 106, 108). However, such dynamics characterize also the traditional model of legal capacity. The model proposed by the CRPD and General Comment No.1 makes at least one step forward. While in current legal capacity legislations doctors and guardians are legally given room to make decisions against the will of the person, the system proposed by the Committee does not contemplate this option, making it at least more laborious for the individuals around the person to abuse their power. Importantly, rather than legal capacity legislation, what really makes people with dementia more exposed to undue influence is the vulnerability deriving from their impairment, and the fact that they are in need of care and completely dependent on people who have either more knowledge or are younger and more resilient than them (69). Therefore, they are inevitably in a position of disadvantage. Current legislations tend to respond to such disadvantage and risk of undue influence by providing “external” safeguards which include mechanisms for reporting suspicious behaviors and inhibiting contact with the person (69, 129), or criminal sanctions in serious cases (116). However, in order to be activated, such safeguards often require articulate procedures, and may be put in place after the undue influence has taken place and some unwanted treatments have already been administered. Moreover, decisions on medical treatment are often made “behind closed doors,” so that abusive behaviors may often go undetected and unreported.

Therefore, what is needed in these cases are “internal” safeguarding mechanisms in which the network of people surrounding the person with dementia and the equilibrium of their conflicting interests makes sure that everyone acts according to the rules and the person's statements are interpreted correctly, without the need for a whistle blower to alert a judicial or administrative authority. The point here is to play with the interests of the various figures around the person with dementia, and make sure that conversations happen in the presence of a set of people, each with symmetrically opposite agendas, that can get in each other's way if some of them oversteps the mark. It's a very old strategy that the law has used to guarantee fairness of procedures and outcomes since its origins and that is behind some of the most successful legal mechanisms for guaranteeing impartiality. It's an idea which is behind, for example, adversarial trials, in which two parties promote symmetrically opposite interests and pull in the opposite direction arriving naturally (if they both do their job well) to a fair outcome.

The same can be done in the field of legal capacity and healthcare decision-making, by using networks of support. For example, the network could include family members or spouse (or an external friend, if family is not available), who should have a good understanding of a person's wishes and biography; their doctor, who gives advice on what could medically be the best solution and is aware of the previous therapeutic pathway of the patient; and an independent advocate, appointed with the specific task of making sure that the will of the person is always center stage. The combined action of these individuals will not create more risks of undue influence because, given their different roles and interests, every member of the group ideally will block attempts from others to exert influence outside their prescribed role. Some of the previously cited shared decision-making experiments provide examples of how these mechanisms can work and how the action of different members of the support network can contribute to attenuate the risk of undue influence. In the study conducted in two nursing homes by Mariani et al., an Italian nurse explained that “during one interview, the family caregiver wanted to focus on a topic that was different from the question addressed to the resident. However, the resident kept repeating the same answer.” Therefore, understanding that the issue was important, healthcare staff intervened and “reassured the resident, who consequently started feeling more at ease, and that she had a leading role in the situation,” and thanks to this intervention the relative also “understood that the topic was important for her mother” (23). These sort of internal and dialog-based safeguards appear more adequate, also in consideration of the fact that undue influence is often well-meaning, and that the line separating support from paternalism can be incredibly fine in practice, coming down to just the choice of words or tone of voice (124). In this regard, what is often needed is more a space for members of the support network to discuss and constantly double-check whether they are “doing it right,” rather than formalized procedures to report misdeeds. Of course, distinguishing between well-meaning empowering influence and undue influence in care relationships will remain at a certain extent a struggle. Much work will still have to be done in analyzing support dynamics and reflecting on how to balance self-determination and assistance in decision-making. However, the CRPD, with its focus on support and the network around the person, puts us on a better ground to have these difficult conversations than the traditional model.

The final major objection raised against the CRPD and General Comment No. 1 in relation to the case of people with dementia is that, by excluding any possibility of substituted decision-making, they do not provide an effective mechanism to deal with what happens in the final stages of dementia, in which the person is often completely unable to communicate with the outside world. In reality, as noted by Series and Nilsson, even the Committee includes, under the category “supported decision-making” practices which are traditionally considered substituted decision-making (2). The mechanism of the “best interpretation of will and preferences” is a form of surrogate decision-making based on the person's presumed will, very similar to what is traditionally referred to as “substituted judgement.” Therefore, despite its strong and apparently uncompromising statements, General Comment No. 1 does not exclude any form of substituted and surrogate decision-making, it merely provides a (re)definition of the concept of supported decision-making, which includes some less paternalistic forms of substitute and surrogate decision-making. Therefore, the criticism raised over the lack of substituted decision-making mechanisms in the model proposed by the Committees seems to be the product of a misunderstanding, due to the fact that many commentators read the General Comment in light of the traditional definitions of supported and substituted decision-making (4–6), not taking into account the different definition adopted by the Committee.

From a strictly technical point of view, this taxonomical choice can seem and it is, to some extent, odd. However, the fact that surrogate and substituted judgement are defined as supported decision-making tools, and integrated in the support spectrum has a practical advantage overcoming some of the limitations emerging in the current system. In the model proposed by the CRPD and the Committee, surrogate decision-making enters into play at the final stage of a support relationship between the person and their relatives, doctors, and carers, in which the individual with dementia and the person who is due to act as a surrogate have already collaborated in making decisions and have exchanged information. In consequence, surrogate decision-makers would not be making decisions for a person the needs of whom they poorly understand, as it often happens in traditional healthcare decision-making models. They would have thoroughly followed the evolution of the person's thoughts and wishes throughout their experience with their condition and would have had the chance to collect more precise information on what the person would want in a certain situation, and thereby be better equipped to make decisions in their place.

Thanks to this, also the application or interpretation of a hypothetical advance directive would be easier. Indeed, the existence of a support relationship before the moment in which the advance directive comes into force, gives the person the possibility to discuss the content of the advance directive with their support person and to update it along the way, as their experience of their condition evolves and their thoughts change, potentially until the very moment before the directive has to be implemented (130–132). In this way, we minimize the risk of ethical dilemmas or interpretive doubts due to the fact that the person seems to perform acts or say things that are in contrast with what expressed in their advance directive years before (132, 133). Also in this case, the time spent by healthcare staff, family members, and carers in talking to the person and supporting them can be used to gather information which will be useful to interpret their anticipated will, and understand the evolution of their thinking from the first draft of their advance directive, to the moment in which their anticipated will needs to be implemented (105, 131, 133–135). Of course, the fact that in the model proposed by General Comment No. 1 there is no distinction between mental capacity and mental incapacity means that advance directives cannot be configured anymore as a single declaration which enters into force at a precise moment in which the person is declared mentally incapable (135). By the same token, this does not mean that advance directives have no part to play in the new system. The Committee refers to advance care planning and advance directives as one (among many) “form[s] of support” which, together, can contribute to reconstruct the will of the person (3). Rather, this instrument takes a different role. In the model proposed by the Committee the advance directive serves as a record over time of the person's will and preferences, and can be used, when and insofar this is necessary, to integrate the indications the person cannot directly express due to their deteriorated condition (130).

Nonetheless, the dogmatically odd terminology suggested by the Committee on the Rights of Persons with Disabilities might create confusion in healthcare and legal professionals and diminish the credibility of Article 12 provisions (6, 32). The adjective “supported” refers specifically to a situation in which an individual is helped by someone else but voices the decision themselves. Including in this notion also cases in which the decision is made *for* the person rather than *by* the person would give the impression of a regulatory model which does not even agree with itself. In addition, there may be cases in which it is the person with dementia who freely wants to delegate the power to make a certain choice to someone else (105). Finally, there might be situations in which the person's will and preferences are expressed in such a way that decisions cannot be made merely on the basis of their best interpretation, but require a certain “creative input” by the support person (8). In all these cases it might be appropriate to partially depart from the indications contained in General Comment No. 1 and distinguish between forms of supported decision-making in the pro*per sense*, and forms of support which entail a certain degree of substitution. In this sense, despite the uncompromising statements of the Committee, I would still find it acceptable if national legislations concede explicitly that in (limited) cases the support relationship may require the use of substitute decision-making mechanisms, perhaps subjecting the use of such mechanisms to more stringent requirements and reporting obligations by the support person, so to avoid that they abuse this option.

## Conclusion

In conclusion, the model of legal capacity proposed by the CRPD, and in particular by General Comment No. 1 by the Committee on the Rights of Persons with Disabilities, is certainly radical and it can appear, at times, utopian, especially because of the way in which some of its claims are formulated. However, once we look more closely to the specifics of this model, we can see that, although it introduces some revolutionary provisions and ideas, it does not depart from the traditional model as strongly as some commentators have argued. Moreover, the changes it proposes seem like a step forward with regard to addressing a series of issues that have emerged in legal and medical practice in relation to cases such as that of healthcare decisions of people with dementia.

In this regard, the CRPD and the Committee propose a more flexible model of legal capacity measures, which avoids artificial distinctions between mental capacity and incapacity, and provides a platform by which the issues faced by people with dementia in deciding on their life and care can be addressed, while preserving the centrality of the person's will. In this way, we avoid the problem of having to binarily assess at which point of the progressive development of the condition they become mentally incapable, a task which has often proven difficult for healthcare professionals. Moreover, it makes sure that doctors, family members, and carers of people with dementia put their best efforts into enabling the person to express their wishes directly through a series of supported decision-making techniques. Finally, also with regard to the final stages of dementia—when it is very difficult if not impossible to communicate with the person—the model proposed by the CRPD and General Comment No. 1 makes sure that next of kin and surrogate decision-makers have all the main resources they need to more adequately make a choice which reflects what the person would have wanted, instead of relying on paternalistic mechanisms based on hard-to-verify assumptions regarding “what would be objectively good” for the person.

Of course, also in the case of the model proposed by the CRPD and the Committee, there is always the risk that the proposed provisions will not be implemented in the correct way, or that the person, because of the situation of vulnerability in which they inevitably find themselves, will be at risk of undue influence or receiving suboptimal support, despite all the mechanisms, strategies and safeguards put in place. Moreover, implementing such a radical change of approach, as that proposed by the CRPD and the Committee, might require, at least in the initial phases, the adoption of compromise solutions in terms of regulatory choices. Therefore, it is important that we keep working on, and discussing, these issues, and finding increasingly effective ways in which we can make the model work in practice. However, it remains the fact that the CRPD and General Comment No. 1 are the best platforms we have to proceed with these conversations and progress in promoting the autonomy and human rights of vulnerable individuals like people with dementia.

## Author's Note

This article originated in the context of the workshop Human Rights and Mental Health, held at the Institute for Medical Ethics and History of Medicine of the Ruhr University Bochum, Germany, on April 1–5, 2019. All opinions expressed in it and possible mistakes remain anyway my responsibility.

## Author Contributions

The author confirms being the sole contributor of this work and has approved it for publication.

## Conflict of Interest

The author declares that the research was conducted in the absence of any commercial or financial relationships that could be construed as a potential conflict of interest.
